# Prevalence of carbapenem-resistant gram-negative bacteria from blood specimen in Adam Malik Hospital Indonesia

**DOI:** 10.1017/ash.2025.136

**Published:** 2025-09-03

**Authors:** Cherry Siregar, Rina Yunita

**Affiliations:** 1 Clinical Microbiology LaboratoryAdam Malik Hospital, Medan, Indonesia; 2 Department of Microbiology, Faculty of MedicineUniversitas Sumatera Utara, Medan, Indonesia

**Keywords:** Carbapenem-resistant, Gram-negative bacteria, Adam Malik Hospital

## Abstract

**Objective:** Carbapenem-resistant Gram-Negative Bacteria (CR-GNB) are a current global concern. CR-GNB in hospitalized patients with bacteremia is a critical health concern due to its high level of resistance to antibiotics and is associated with high mortality rates. This study aims to identify the prevalence of CR- GNB from blood specimens of patients in Adam Malik Hospital. **Method:** A retrospective cross-sectional study was carried out on blood specimens of patients received at the Clinical Microbiology Laboratory Adam Malik Hospital from January 2023 to December 2023. The bacterial isolates were identified using BD™ Bruker MALDI Biotyper and the susceptibility of the isolates to various antimicrobial agents was tested using the automated antimicrobial susceptibility tests. We performed a descriptive statistical analysis of the Gram-negative bacterial growth from blood specimens and antimicrobial susceptibility against each bacterial isolate. **Results:** There were 939 bacterial isolates obtained from blood culture, and 57% (534/938) were Gram-negative. The most prevalent Gram-negative bacteria were *Klebsiella pneumoniae* (22.09%), *Escherichia coli* (19.84%), *Pseudomonas aeruginosa* (9.20%), and *Acinetobacter baumannii* (7.89%). Among all the Gram-negative isolates, 18,1% were carbapenem-resistant. The most prevalent Gram-negative bacteria that are resistant to carbapenems were *A. baumannii* (56.4%);22/39), *K. pneumoniae* (25.2%; 27/107), *P. aeruginosa* (22.7%; 10/44), *Proteus mirabilis* (20%; 1/5) and *Enterobacter cloacae* (18.6%);8/43). The highest antimicrobial susceptibility for the most prevalent CR-GNB were amikacin for *E. cloacae*, *P. aeruginosa* and *A. baumannii* (87,5%, 60 %; 40%; respectively), trimethoprim-sulfamethoxazole for *K. pneumonia* (66,7%), tigecycline for *E. cloacae* (87,5%). **Conclusion:** The prevalence of CR-GNB from blood specimens in Adam Malik Hospital was 18,1%. The most common CR-GNB isolates were *Acinetobacter baumannii, K. pneumoniae, P. aeruginosa, and Enterobacter cloacae*. The infection control program is a critical action to prevent the transmission of CR-GNB, particularly in hospital settings.

